# ECG Signal Classification Using Deep Learning Techniques Based on the PTB-XL Dataset

**DOI:** 10.3390/e23091121

**Published:** 2021-08-28

**Authors:** Sandra Śmigiel, Krzysztof Pałczyński, Damian Ledziński

**Affiliations:** 1 Faculty of Mechanical Engineering, UTP University of Science and Technology in Bydgoszcz, 85-796 Bydgoszcz, Poland; 2 Faculty of Telecommunications, Computer Science and Electrical Engineering, UTP University of Science and Technology in Bydgoszcz, 85-796 Bydgoszcz, Poland; krzysztof@palczynski.com.pl (K.P.); damian.ledzinski@utp.edu.pl (D.L.)

**Keywords:** ECG signal, classification, PTB-XL, deep learning

## Abstract

The analysis and processing of ECG signals are a key approach in the diagnosis of cardiovascular diseases. The main field of work in this area is classification, which is increasingly supported by machine learning-based algorithms. In this work, a deep neural network was developed for the automatic classification of primary ECG signals. The research was carried out on the data contained in a PTB-XL database. Three neural network architectures were proposed: the first based on the convolutional network, the second on SincNet, and the third on the convolutional network, but with additional entropy-based features. The dataset was divided into training, validation, and test sets in proportions of 70%, 15%, and 15%, respectively. The studies were conducted for 2, 5, and 20 classes of disease entities. The convolutional network with entropy features obtained the best classification result. The convolutional network without entropy-based features obtained a slightly less successful result, but had the highest computational efficiency, due to the significantly lower number of neurons.

## 1. Introduction

According to publicly available reports, cardiovascular disease remains the leading cause of mortality worldwide [1]. One of the main causes of cardiovascular diseases is cardiac arrhythmia, in which the heartbeat deviates from typical beating patterns [2]. However, there are many types of irregular heartbeat. Accurate classification of heart disease types can aid in diagnosis and treatment [3].

An electrocardiogram (ECG) is a widely used, reliable, noninvasive approach to diagnosing cardiovascular disease. The standard ECG consists of 12 leads [4]. Traditionally, ECG results are manually interpreted by cardiologists based on a set of diagnosis criteria and experience. However, manual interpretation is time consuming and requires skill. Incorrectly interpreted ECG results may give rise to incorrect clinical decisions and lead to a threat to human life and health. With the rapid development of ECG and, at the same time, an insufficient number of cardiologists, the accurate and automatic diagnosis of ECG signals has become an interesting research topic for many scientists.

Over the past decade, numerous attempts have been made to identify a 12-lead clinical ECG, largely on the basis of the availability of large, public, open-source ECG data collections. Previous literature on ECG databases has shown a methodological division: signal processing and machine learning [5,6]. On the one hand, digital signal processing methods mainly include low- or high-pass filters, fast Fourier transform, and wavelet transform [7]. In this area, many algorithms are based on three processes: feature extraction, feature selection, and classification [8]. On the other hand, an alternative method is the application of machine learning methods. Such an application would primarily focus on the automatic recognition of patterns that classify various disease entities, a method that is gaining greater importance in medical practice.

Algorithms known as deep neural networks have become particularly important in the last five years. Deep learning models have proven to be useful in increasing the effectiveness of diagnoses of cardiovascular diseases using ECG signals. By using the cascade of heterogeneous layers of neural networks to gradually extract increasingly high-level features, they lead to ever-improving neural networks built on their basis. Deep neural networks are reaching their zenith in various areas where artificial intelligence algorithms are applied.

In recent years, machine learning models have given rise to huge innovations in many areas, including image processing, natural language processing, computer games, and medical applications [9]. To date, however, the lack of adequate databases, well-defined assessment procedures, and unambiguous labels identifying signals has limited the possibilities for creating an automatic interpretation algorithm for the ECG signal. Known databases provided by PhysioNet, such as the MIT-BIH Arrhythmia Database and the PTB Diagnostic ECG Database, were deemed insufficient [10,11]. Data from single, small, or relatively homogeneous datasets, further limited by a small number of patients and rhythm episodes, prevented the creation of algorithms in machine learning models.

The work of the PhysioNet/Computing in Cardiology Challenge 2020 project to develop an automated ECG classifier provided an opportunity to address this problem by adding data from a wide variety of sources. Among these, there are numerous works, including the development of a comprehensive deep neural network model for the classification of up to 27 clinical diagnoses from the electrocardiogram. The authors of one of these achieved results, using the ResNet model, at the level of AUC = 0.967 and ACC = 0.43 in their study [12]. A similar approach was proposed [13], using the SE_ResNet model to improve the efficiency of the classification of various ECG abnormalities. Others, focusing on the comparative analysis of the recently published PTB-XL dataset, assessed the possibility of using convolutional neural networks, in particular those based on the ResNet and Inception architectures [14]. A different approach in the classification of cardiovascular diseases was demonstrated by the authors of a work [15] related to the detection of QRS complexes and T & P waves, together with the detection of their boundaries. The ECG classification algorithm was based on 19 classes. Features were extracted from the averaged QRS and from the intervals between the detected points.

The 12-lead ECG deep learning model found its reference mainly to ECG diagnosis in the automatic classification of cardiac arrhythmias. A deep learning model trained on a large ECG dataset was used with a deep neural network [16] based on 1D CNN for automatic multilabel arrhythmia classification with a score of ACC = 0.94 − 0.97. The authors of this study also conducted experiments on single-lead ECG with an analysis of the operation of every single lead. The subject of arrhythmia classification is also of interest to other authors [17], where, with the use of long-short term memory (LSTM), a model with an LSTM score of 0.6 was proposed. The choice of ECG for arrhythmia detection was undertaken by the authors of the paper [18], where they designed a computer-aided diagnosis system for the automatic diagnosis of four types of serious arrhythmias. In this approach, the ECG was analyzed using thirteen nonlinear features, known as entropy. The features extracted in this way were classified using ANOVA and subjected to automated classification using the K-nearest neighbor and decision tree classifiers. The obtained results were for KNN − ACC = 93.3% and DT − ACC = 96.3%. Various deep learning models for the examination of the ECG signal have also been proposed for atrial fibrillation, obtaining the result of ACC = 0.992 [19]. It is worth noting that the presented model successfully detected atrial fibrillation, and the tests were carried out with the use of various ECG signals. Attempts to investigate cardiac arrhythmias and cardiovascular diseases were also carried out in a new convolutional neural network [9] with a nonlocal convolutional block attention module (NCBAM), which focused on representative features along space, time, and channels. For the classification problem of ECG arrhythmia detection, the authors obtained AUC = 0.93. The approach to convolutional neural networks, the possibilities and usability of tools, and the analysis of biomedical signals were also proposed by the authors of other papers [20]. The research included the implementation of a multilabel classification algorithm with the use of machine learning methods based on a CNN. The work described the details of the algorithm necessary for reconstruction and presented limitations and suggestions for improvement. A different approach to the ECG signal was presented by the authors of [21], where the focus was instead placed on processing the ECG signal, data sampling, feature extraction, and classification. They used a deep learning class model with gated recursive complex (GRU) and extreme learning machine (ELM) to recognize the ECG signal.

The aim of the study was to check the effectiveness of multiclass classification of ECG signals with the use of various neural network architectures. An additional aim was to test the effectiveness of very light nets for classification. A novelty in the article is the combination of a neural network with entropy-based features.

## 2. Materials and Methods

### 2.1. PTB-XL Dataset

**Figure 1:**
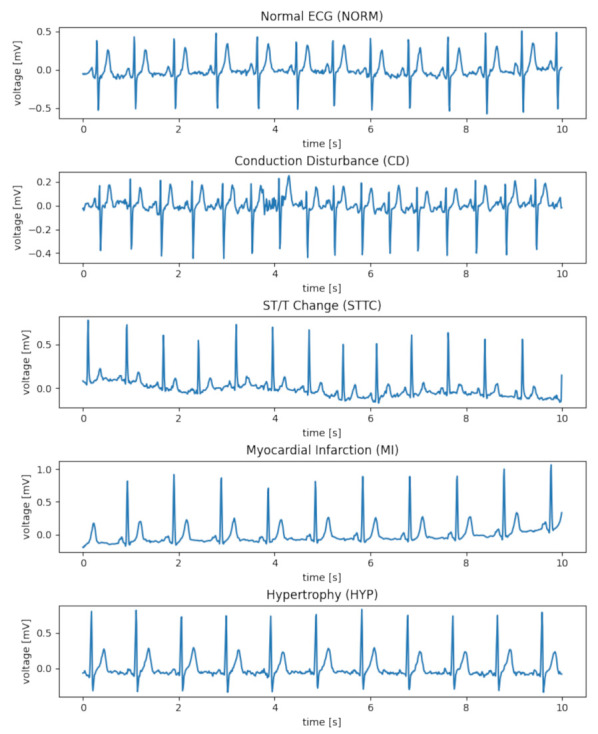
Examples of rhythm ECG signals.

In this article, data from the PTB-XL ECG database were used [11]. The PTB-XL database is a clinical ECG dataset of unprecedented size, with changes applied to evaluate machine learning algorithms. The PTB-XL ECG dataset contains 21,837 clinical 12-lead ECGs from 18,885 patients of 10 s in length, sampled at 500 Hz and 100 Hz with 16 bit resolution. Figure 1 shows examples of rhythms, consistent with the data contained in Table 1, which were used in the work. Among them there are examples of the following ECG signals: NORM—normal ECG, CD—myocardial infarction, STTC—ST/T change, MI—conduction disturbance, HYP—hypertrophy.

The PTB-XL database is gender balanced. The data included were derived from 52% males and 48% females, ranging in age from 2 to 95 years (median 62). The data were enriched with additional information about the patient (age, sex, height, weight). Each ECG by the authors of the dataset was classified into one or more of 23 diagnostic subclasses in 5 diagnostic classes, or into classes that are not diagnostic classes. Each class was assigned a probability. Classes are marked according to the standard with the codes SCP_ECG.

The research methodology included classification studies carried out in 3 categories of binary classifications, where the classes were NORM (healthy patient) and all other classes (sick patient), where 5 diagnostic classes were used and where 20 diagnostic subclasses were used.

**Figure 2:**
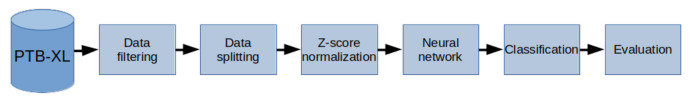
General overview diagram of the method.

The research methodology was as follows (Figure 2): Data from the PTB-XL database were filtered and then divided into training, validation, and test groups. These data were then normalized and used as inputs for the neural networks that were examined. The network performed a classification. The signal class was obtained as an output, and this was then subjected to evaluation.

During the filtering stage, a set of 21,837 ECG records from the PTB-XL database was included in the simulation. ECGs not classified into diagnostic classes were filtered from the dataset. Subsequently, the ECGs in which the probability of classification was less than 100% were filtered out. In the next stage, ECGs were filtered out of those subclasses whose presence in the dataset was less than 20. A sampling frequency of 100 Hz was selected for the study, with 10 s as the length.

The dataset was divided into training, validation, and test sets in proportions of 70%, 15%, and 15%, respectively. The training set was used to train the network; the validation set was used to select the model; the test set was used to test the network’s effectiveness.

**Figure 3:**
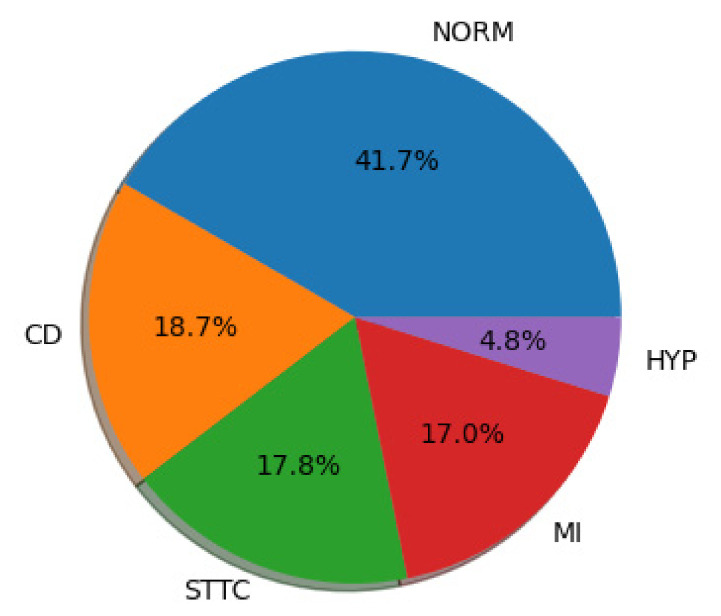
Diagnostic classes used in the study.

As a result of the above activities, a total of 17,232 ECG records were used for the experimental analysis (Figure 3).

A detailed summary of the size of the individual classes used in the study and resulting from the above-described activities on the basis of PTB-XL is presented in Table 1 and Table 2. The tables show the number of individual records used in the study, assigned to the appropriate diagnostic classes and subclasses defining cardiovascular diseases sorted by number of records.

### 2.2. Designed Network Architectures

This research compared three neural networks (convolutional network, SincNet, convolutional network with entropy features) in terms of the correct classification of the ECG signal. The research consisted of the implementation and testing of the proposed models of the neural networks. Cross-entropy loss as a loss function was applied to all networks.

The artificial neural networks proposed in this article were based on layers performing one-dimensional convolutions. This is a state-of-the-art solution in signal processing using deep learning due to its ability to extract features based on changes in consecutive samples, while simultaneously being faster and easier to train than recurrent layers such as LSTMs. The convolutional networks described in this article also contain residual connections between convolutional layers as described in [22]. These shortcut connections eliminate the so-called vanishing gradient problem and increase the capacity of models for better representation learning.

The networks were trained using the Adam optimizer as described in [23]. The optimizer trained the neural network using mini-batches of 128 examples in one pass. The learning rate was set at 0.001 at the beginning of the training and was later adjusted to 0.0001 to perform final corrections before ending the training. To prevent overfitting, early stopping was employed as described in [24]. The training of the neural network was stopped as soon as the network was unable to obtain better results on the validation dataset. This was to prevent overfitting. Following testing, the neural network was trained on the test dataset.

The tests were carried out using hardware configurations on a dual-Intel Xeon Silver 4210R, 192 GB RAM, and Nvidia Tesla A100 GPU. In this research, PyTorch and Jupyter Lab programming solutions were used for the implementation of the neural networks.

#### 2.2.1. Convolutional Network

**Figure 4:**
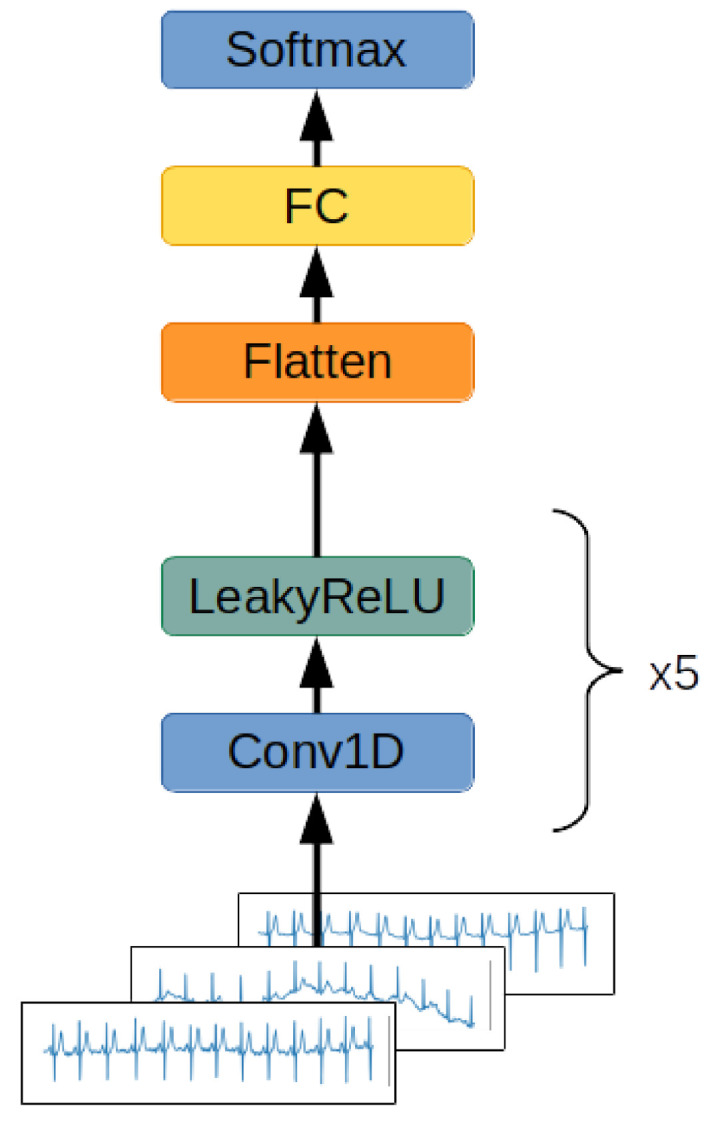
Convolutional network architecture. A twelve-channel ECG signal is passed through five subsequent one-dimensional convolutional layers with the LeakyReLU activation function. The results of the computation are flattened to the format of a one-dimensional vector. The results of the calculation are processed by a fully connected layer with a softmax activation function. The output value is a one-dimensional vector describing the probability distribution of the input signal belonging to each of the defined classes.

The first network examined is presented in Figure 4. It consists of five layers of one-dimensional convolutions with LeakyReLU activation functions and one fully connected layer with a softmax activation function. The network accepts ECG signals consisting of 12 channels containing 1000 samples each as inputs and outputs a class distribution vector normalized by application of the softmax function. The network determines the class to which an input signal belongs by determining the index of the vector maximum value. The class represented by this index is considered as a class of the input signal.

LeakyReLU was used instead of basic ReLU to preserve gradient loss in neurons outputting negative values. The coefficient describing a negative slope was set to 0.01; thus, the activation function can be described by the equation below:

(1) $$f\left(x\right)=\left\{\begin{array}{cc} 0.01x & \;\mathrm{for}\;x<0\\ x & \;\mathrm{for}\;x\geq 0 \end{array}\right.$$

This configuration was used in every network proposed in this article.

This architecture was tested on both the normalized signal taken from the dataset without any other transformations and a spectrogram, and the results obtained from the former were better than from the latter. The network computing the spectrogram interpreted each spectrogram as a multichannel one-dimensional signal. Each of the twelve signals’ spectrograms was processed by five one-dimensional convolutional blocks with the LeakyReLU activation function. The results of the convolutions where aggregated by performing adaptive average pooling. Afterwards, the results of pooling were flattened to the format of a one-dimensional vector and processed by a fully connected layer with a softmax activation function, and the output was used as a vector describing the probability distribution of the input signals belonging to each of the defined classes.

This is a simplified architecture designed to achieve both better computation time and memory storage efficiency. This network design has only 6 layers and, depending on the number of classes in classification, has just 8882 weights for binary classification and 11,957 weights for detecting 5 different classes of signal. The last segment of the network is a fully connected layer, which has a number of neurons equal to the quantity of possible classes to which the signal may belong. As a result, the more granular the classification process is, the more neurons are required, which increases the number of total weights in the network. The addition of residual connections did not increase the performance of the network significantly, but enlarged the quantity of parameters and computational steps required to process the signal.

#### 2.2.2. SincNet

The second examined network uses the SincNet layers described in [25]. SincNet layers are designed for the extraction of low-level features from a raw signal’s data samples. SincNet layers train “wavelets” for feature extraction by performing convolution on the input signal:

(2) y[n]=x[n]·g[n,θ]

where *n* is the index of the probe and θ are the parameters of the wavelets determined during training. The wavelet function *g* is described with the equation:

(3) $$g\left[n,f_{1},f_{2}\right]=2f_{2}sinc\left(2\pi f_{2}n\right)-2f_{1}sinc\left(2\pi f_{1}n\right)$$

where sinc function is defined as:

(4) $$sinc\left(x\right)=\frac{sin(x)}{x}$$

$f_{1}$ and $f_{2}$ are the cutoff frequencies determined by the SincNet layer during the training phase and form a set of trainable parameters θ:

(5) $$\theta =\{\left(f_{i,1},f_{i,2}\right)|i\in C^{+}\cap i\leq l\}$$

where *l* is the number of wavelets in the SincNet layer.

The pair of filters ($f_{1}$,$f_{2}$) are initialized using the frequencies used for calculation of Mel-frequency cepstral coefficients [26].

**Figure 5:**
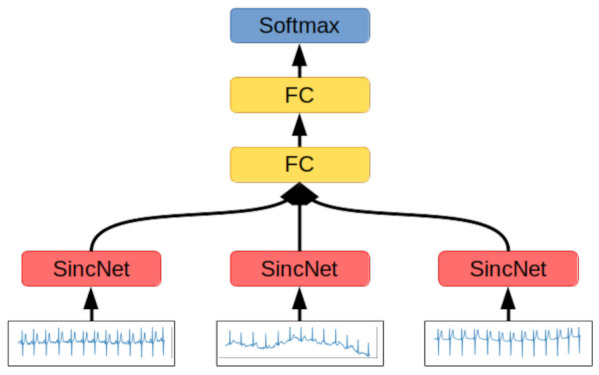
SincNet-based network architecture. Each channel of the 12-channel ECG signal is processed by a dedicated SincNet block. The results of each block are concatenated, flattened to the format of a one-dimensional vector, and used as an input for two subsequent fully connected layers, with LeakyReLU and softmax activation functions, respectively. The output value is a one-dimensional vector describing the probability distribution of the input signal belonging to each of the defined classes.

SincNet layers are designed to interpret only the signal’s singular channel at once, so the second network’s architecture consists of a subnetwork using a SincNet layer, which encodes each signal’s channel separately. The features extracted by the subnetwork are concatenated into one feature vector, which is fed to a block of fully connected layers. The softmax layer serves the role of the output classification layer, while the SincNet subnetwork consists of the SincNet layer adjusting the wavelets to the raw signal, two convolutional layers with LeakyReLU activation functions and layer normalizations, and three fully connected layers with batch normalization and LeakyReLU activation functions (Figure 5).

#### 2.2.3. Convolutional Network with Entropy Features

**Figure 6:**
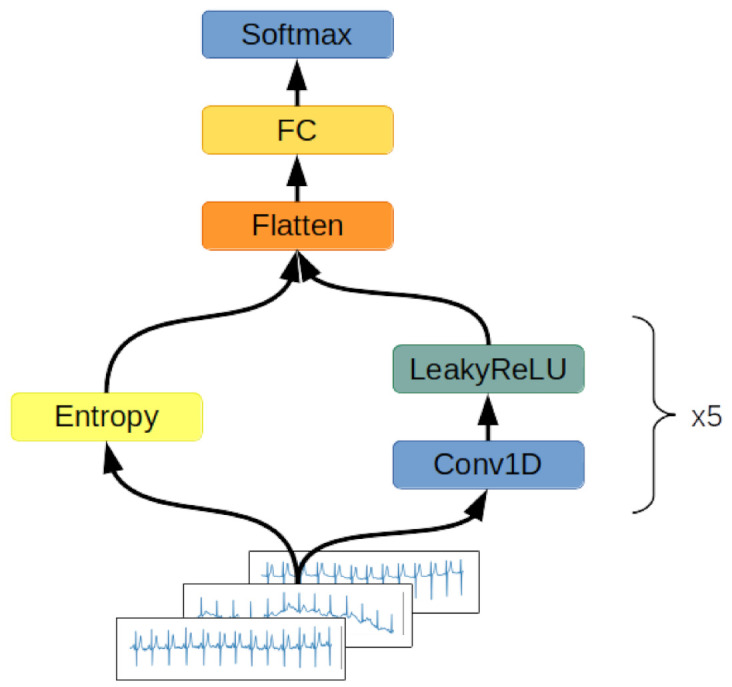
Convolutional network with the entropy features’ block architecture. The computational graph of the network is made up of two branches. In the first branch, a twelve-channel ECG signal is passed through five subsequent one-dimensional convolutional layers with the LeakyReLU activation function. In the second branch, the input signal is used to compute the vector of entropies for every channel of the signal. The results of the computations from both branches are concatenated and flattened to the format of a one-dimensional vector. The results of the calculation are processed by a fully connected layer with softmax activation function. The output value is a one-dimensional vector describing the probability distribution of the input signal belonging to each of the defined classes.

The third network examined is presented in Figure 6. This network is an extended variant of the convolutional network. The network processes the ECG signal, and the values of the entropies are calculated for every channel of the signal. These entropies are:

- Shannon entropy—the summation of the informativeness of every possible state in the signal by measuring its probability. As a result, Shannon entropy is the measurement of the spread of the data [27];
- Approximate entropy—the measurement of series regularity. It provides information on how much the ECG fluctuates and its predictability [28];
- Sample entropy—an improvement on approximate entropy due to the lack of the signal length’s impact on the entropy computations [28];
- Permutation entropy—the measurement of the order relations between ECG samples. This quantifies how regular and deterministic the signal is [29];
- Spectral entropy—the quantification of the energy spread uniformness across the frequency spectrum [30];
- SVD entropy—the measurement of how possible the dimensionality reduction of time series matrix is through factorization using the eigenvector approach;
- Rényi entropy—the generalization of the Shannon entropy by introducing the fractal order of the subsequent informativeness of each signal’s state [31];
- Tsallis entropy—the generalization of the Boltzmann–Gibbs entropy, able to detect long-term memory effects on the signal [32];
- Extropy—the measurement of the amount of uncertainty represented by the distribution of the values in the observed ECG signal [33].

Granelo-Belinchon et al., in their article [34], stated that the tools of information theory can be straightforwardly applied to any nonstationary time process when considering small chunks of data spanning a short enough time range, allowing a slow evolution of higher-order moments to be neglected. The augmented Dickey–Fuller test has been conducted on ten-second-long training chunks of signals to determine the momentary stationarity of ECG signals. It turned out that 89.5% of tested signals were deemed stationarity in this small period of time, allowing the use of entropy methods for their interpretation.

The artificial neural network consists of two blocks: convolutional and fully connected. In the first step, a raw ECG signal is encoded by a convolutional block formed by five one-dimensional convolutional layers with the LeakyReLU activation function. Each layer has a stride parameter equal to 2 to reduce the number of samples representing the time vector. Each layer also has a residual connection with the original, raw signal. Because of the signal’s sample reduction due to the applied stride parameter, the ECG signal for each step of the residual connection is shrunk by average pooling with a window size of 2.

The encoded raw ECG signal is concatenated with the values of the entropies of every channel. Such a feature vector is fed to three fully connected layers with LeakyReLU activation functions in the first two and a softmax function in the last layer. The result of the softmax function is the output vector of the network and is used in order to classify the signal. For regularization purposes, there was a dropout with a chance of zeroing the input equal to 20% applied before each layer. The dropout was turned off during the network’s evaluation.

### 2.3. Metrics

The neural networks were evaluated using the metrics described below. For the purpose of the simplicity of the equations, certain acronyms were created, as follows: TP—true positive, TN—true negative, FP—false positive, FN—false negative. The metrics used for the network evaluation are:

- Accuracy: Acc = (TP + TN)/(TP + FP + TN + FN);
- Precision = TP/(TP + FP);
- Recall = TP/(TP + FN);
- F1 = 2 * precision * recall/(precision + recall);
- AUC—area under the curve, ROC—area under the receiver operating characteristic curve. The ROC is a curve determined by calculating TFP = true positive rate = TP/(TP + FN) and FPR = false positive rate = FP/(TN + FP). The false positive rate describes the x-axis and the true positive rate the y-axis of a coordinate system. By changing the threshold value responsible for the classification of an example as belonging to either the positive or negative class, pairs of TFP-FPR are generated, resulting in the creation of the ROC curve. The AUC is a measurement of the area below the ROC curve;
- Total Params—number of neurons in the network. The smaller this number, the better, as less computation is required in order to perform classification.

## 3. Results

The results of the networks based on the convolutional network, SincNet, and the convolutional network with entropy features are summarized in Table 3, Table 4 and Table 5. With the recognition of two classes, the network based on the convolutional network achieved 88.2% ACC and with five classes 72.0% ACC. Similarly, the network based on SincNet achieved 85.8% ACC with the recognition of two classes and 73.0% with the recognition of five classes. The network based on the convolutional network with entropy features achieved 89.82% ACC with the recognition of two classes and 76.5% with the recognition of five classes. The network based on the convolutional network turned out to be slightly better than that based on SincNet. The situation changed with the recognition of 20 classes, where SincNet turned out to be slightly more effective. However, the network based on the convolutional network with entropy features turned out to be the best in all cases. It is worth noting that, depending on the number of recognized classes, the convolutional network had 200–600-times less weight than the SincNet-based network, which means it is much lighter. Adding entropy-based features to the convolutional network increases its weight two- to seven-fold. The convolutional neural network with entropy features achieved the highest accuracy in every classification task, scoring 89.2%, 76.5%, and 69.8% for 2, 5, and 20 classes, respectively. The basic convolutional network achieved better accuracy than SincNet during the classification of two classes (healthy/sick), but SincNet performed better on the classification of five and twenty classes. As described by Ravanelli et al. in [25], the neural network was designed to process the human voice without any data preprocessing and did so successfully according to the authors. However, the results of its usage on ECG signals are far from ideal, as presented in Table 3, Table 4 and Table 5.

Figure 7, Figure 8, Figure 9, Figure 10, Figure 11, Figure 12, Figure 13, Figure 14 and Figure 15 show the confusion matrices of the results of the evaluated networks.

In all cases of the evaluated networks, the NORM class obtained the highest value, which resulted from the large number of ECG recordings in this class.

## 4. Discussion

This paper presented a new model of convolutional neural networks, optimized to limit the computational and memory complexity for ECG recognition and classification of cardiovascular diseases. The research was carried out using a CNN network based on the convolutional network, which is relatively light and yields good results. The advantage of this approach is the possibility of using it on mobile and embedded devices, such as a Raspberry Pi or smartphone graphics cards.

The application of additional entropy-based features significantly improved the results. Such a solution also increased the weight of the network several times, however. As a result, in applications where a very light network is needed, a compromise between weight and accuracy should be sought.

SincNet is a promising solution, but due to being designed to work with the human voice, it does not cope well with ECG signals in its original format. This results from the use of a set of initialization frequencies used in the computation of the Mel-frequency cepstral coefficients that are adapted to the spectral characteristics of the human voice. In the future, it would be worth considering the possibility of adapting SincNet to work with ECG.

The authors were unable to obtain better results due to the issue of overfitting on the training dataset. It was presumed that the addition of customized features may further boost the performance. The authors plan to investigate this claim in their next work.

Sampling determines the amount of measurements used to describe the signal. By changing the sampling, the signal is described by either more or fewer samples, whereas a stack of convolutional layers processes a fixed number of measurements in one context window. As a result, through a modification of the signal sampling, the network may either come to focus on more global features by reducing the amount of samples describing the signal or increase its attention to the details by increasing the measurements per signal.

Interpreting signals with different samplings may prove beneficial. In this work, we used only signals encoding 10 s of experiment on 1000 samples. It may well be the case that a network simultaneously interpreting a signal sampled with frequencies of 500 samples per second, 100 samples per second, and 50 samples per second will return better results. This is because signals sampled at lower frequencies can have entire ECG waves interpreted by one convolutional block, while signals sampled more frequently provide more detailed series for the extraction of features encoded by a small part of an ECG wave.

The proposed network based on a convolutional network is relatively uncomplicated. It is likely that better results could be obtained with the use of Inception models. This model uses heterogeneous subnets to improve the result. It is comparable to the case of wavelet transform, which may prove to be more advantageous than the use of fast Fourier transform. According to the authors, the proposed solution could be used in small devices for continuous monitoring of ECG signals, for example to alert about anomalies and make an initial diagnosis or support a doctor in this.

The authors assumed that a network’s performance may be improved with a manageable cost increase by expanding its architecture with Inception-style heterogeneous subnetworks with varying kernels and poolings. The authors intend to investigate this assumption in their future work.

The authors further assumed that the integration of SincNet layers for low-level feature extraction in the first step of signal processing with the successful implementation of the first network based on convolutional layers may prove a benefit. The authors intend to investigate this assumption in their future work.

## 5. Conclusions

This study presented the capability of convolutional neural networks in the classification of heart diseases by the examination of ECG signals. The network proposed by the authors is both accurate and efficient as it is lightweight, allowing it to be computed on nonspecialized devices. The application of entropy-based features proved beneficial due to the improvements in the accuracy of heart disease classification. Entropy-based features are promising additions to data preprocessing that may prove beneficial in other signal-processing-related tasks.

## Figures and Tables

**Figure 7:**
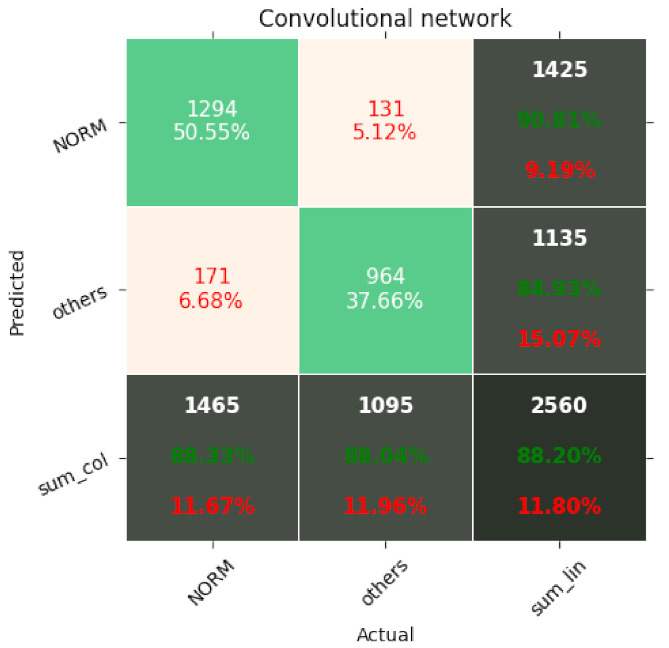
Confusion matrix of results for 2 classes for the convolutional network.

**Figure 8:**
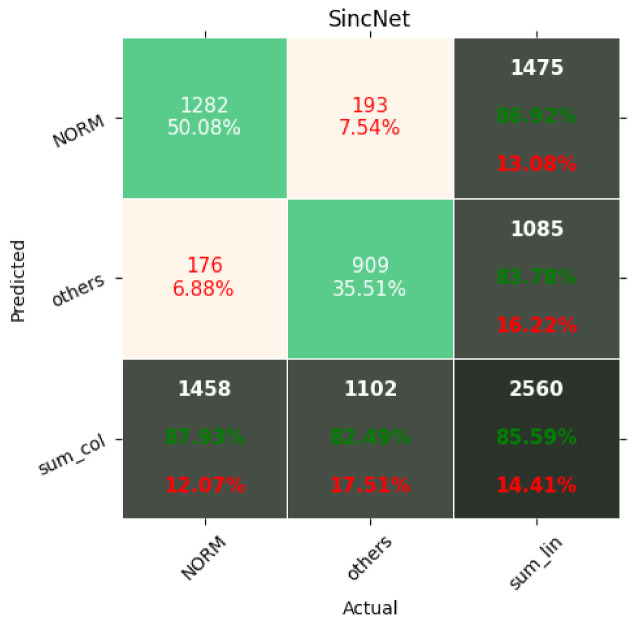
Confusion matrix of results for 2 classes for SincNet.

**Figure 9:**
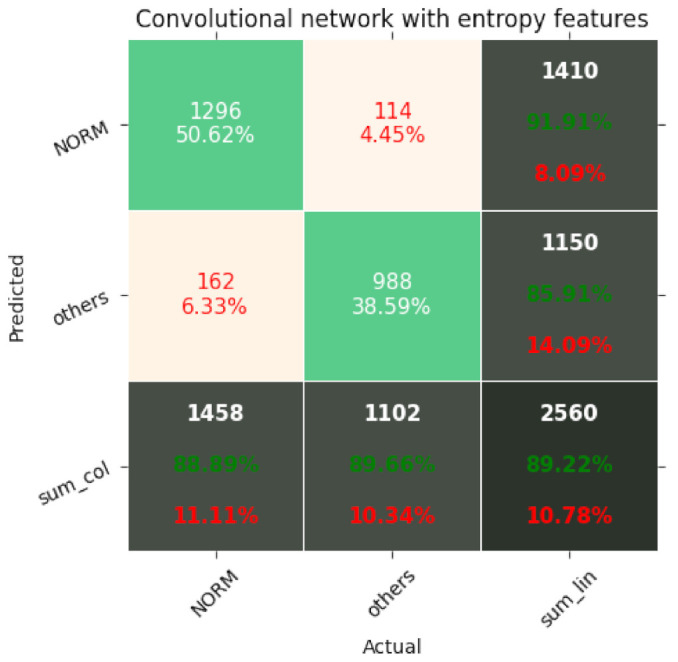
Confusion matrix of results for 2 classes for the convolutional network with entropy features.

**Figure 10:**
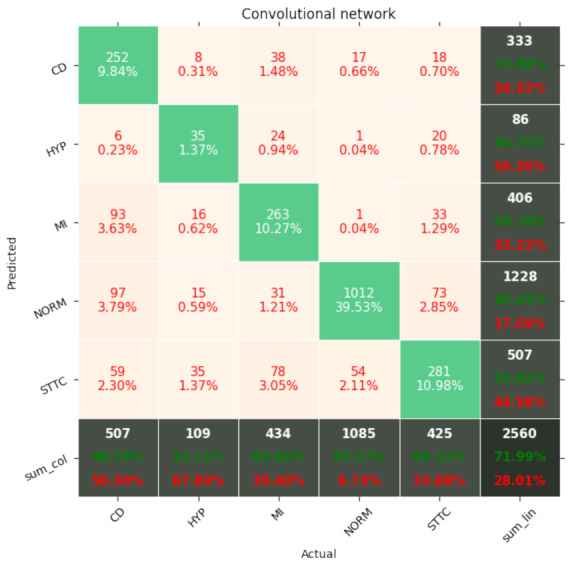
Confusion matrix of results for 5 classes for the convolutional network.

**Figure 11:**
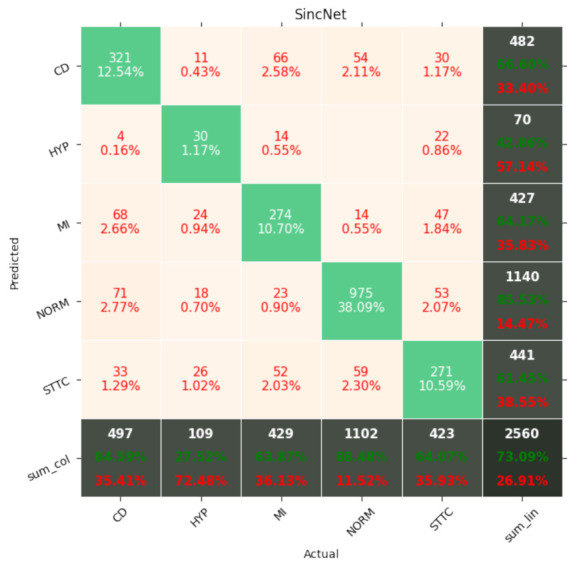
Confusion matrix of results for 5 classes for SincNet.

**Figure 12:**
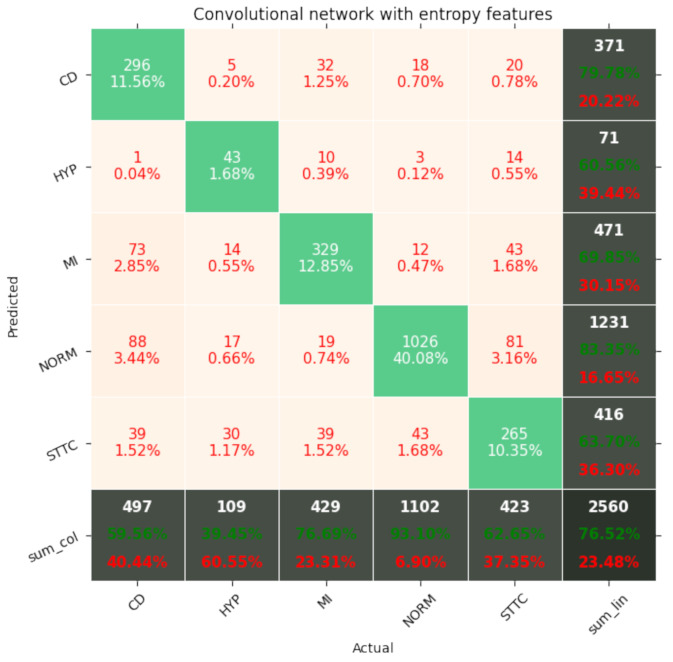
Confusion matrix of results for 5 classes for the convolutional network with entropy features.

**Figure 13:**
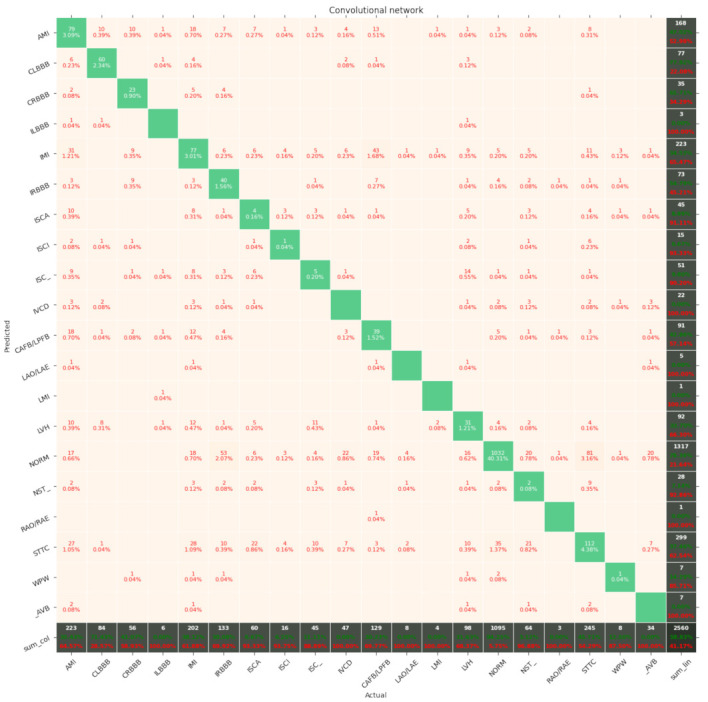
Confusion matrix of results for 5 classes for the convolutional network.

**Figure 14:**
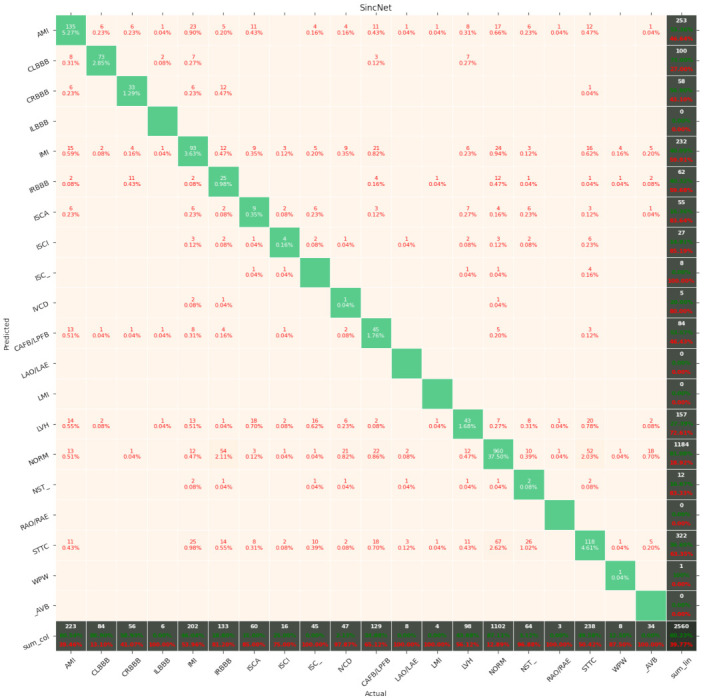
Confusion matrix of results for 5 classes for SincNet.

**Figure 15:**
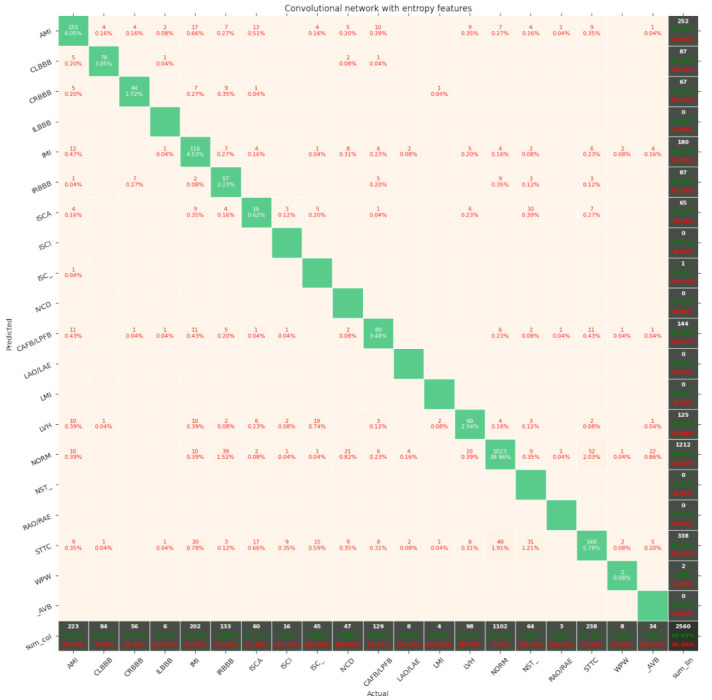
Confusion matrix of results for 5 classes for the convolutional network with entropy features.

**Table 1:** The numbers of individual classes.

Number of Records	Class	Description
7185	NORM	Normal ECG
3232	CD	Myocardial Infarction
3064	STTC	ST/T Change
2936	MI	Conduction Disturbance
815	HYP	Hypertrophy

**Table 2:** Numbers of individual subclasses.

Number of Records	Subclass	Class	Description
7185	NORM	NORM	Normal ECG
1713	STTC	STTC	Non-diagnostic T abnormalities, suggests digitalis effect, long QT interval, ST-T changes compatible with ventricular aneurysm, compatible with electrolyte abnormalities
1636	AMI	MI	Anterior myocardial infarction, anterolateral myocardial infarction, in anteroseptal leads, in anterolateral leads, in lateral leads
1272	IMI	MI	Inferior myocardial infarction, inferolateral myocardial infarction, inferoposterolateral myocardial infarction, inferoposterior myocardial infarction, in inferior leads, in inferolateral leads
881	LAFB/LPFB	CD	Left anterior fascicular block, left posterior fascicular block
798	IRBBB	CD	Incomplete right bundle branch block
733	LVH	HYP	Left ventricular hypertrophy
527	CLBBB	CD	(Complete) left bundle branch block
478	NST_	STTC	Nonspecific ST changes
429	ISCA	STTC	In anterolateral leads, in anteroseptal leads, in lateral leads, in anterior leads
385	CRBBB	CD	(Complete) right bundle branch block
326	IVCD	CD	Nonspecific intraventricular conduction disturbance
297	ISC_	STTC	Ischemic ST-T changes
204	_AVB	CD	First-degree AV block, second-degree AV block, third-degree AV block
147	ISCI	STTC	In inferior leads, in inferolateral leads
67	WPW	CD	Wolff–Parkinson–White syndrome
49	LAO/LAE	HYP	Left atrial overload/enlargement
44	ILBBB	CD	Incomplete left bundle branch block
33	RAO/RAE	HYP	Right atrial overload/enlargement
28	LMI	MI	Lateral myocardial infarction

**Table 3:** The results of the convolutional network.

Number of Classes	Acc	Avg Precision	Avg Recall	Avg F1	Avg AUC	Total Params
**2**	0.882	0.879	0.882	0.88	0.953	8882
**5**	0.72	0.636	0.602	0.611	0.877	11,957
**20**	0.589	0.259	0.228	0.238	0.856	27,332

**Table 4:** The results of SincNet.

Number of Classes	Acc	Avg Precision	Avg Recall	Avg F1	Avg AUC	Total Params
**2**	0.858	0.855	0.854	0.855	0.93	6,109,922
**5**	0.73	0.666	0.589	0.6	0.884	6,109,922
**20**	0.593	0.287	0.269	0.262	0.807	6,269,204

**Table 5:** The results of the convolutional network with entropy features.

Number of Classes	Acc	Avg Precision	Avg Recall	Avg F1	Avg AUC	Total Params
**2**	0.892	0.889	0.893	0.891	0.96	58,178
**5**	0.765	0.714	0.662	0.68	0.910	58,259
**20**	0.698	0.355	0.339	0.332	0.815	58,664

## Data Availability

The data presented in this study are available upon request from the corresponding author.
